# RETRACTED: Jeong et al. A Phase III Head-to-Head Study to Compare the Efficacy and Safety of Fexuprazan and Esomeprazole in Treating Patients with Erosive Esophagitis. *J. Clin. Med.* 2024, *13*, 3262

**DOI:** 10.3390/jcm13216388

**Published:** 2024-10-25

**Authors:** Yuchul Jeong, Beom Jun Lee, Se-Hyeon Han

**Affiliations:** 1 Department of Internal Medicine, Chungna Good Hospital, Incheon 22738, Republic of Korea; 2 St. Mary’s Best ENT Clinic, Seoul 08849, Republic of Korea; 3 Department of Companion Animal Industry, College of Health Science, Honam University, Gwangju 62399, Republic of Korea

The *Journal of Clinical Medicine* retracts the article entitled “A Phase III Head-to-Head Study to Compare the Efficacy and Safety of Fexuprazan and Esomeprazole in Treating Patients with Erosive Esophagitis” [1].

Following publication, concerns were brought to the attention of the Editorial Office regarding an overlap between this publication [1] and a previous publication [2], produced by a different authorship group.

Adhering to our complaint’s procedure, an investigation was conducted by the Editorial Office and the Editorial Board that confirmed a significant similarity between the data presented in the two publications [1,2]. As a result, the Editorial Board consider this publication [1] to be redundant, and therefore have decided to retract it as per MDPI’s retraction policy (https://www.mdpi.com/ethics#_bookmark30, accessed on 22 October 2024).

This retraction was approved by the Editor-in-Chief of the *Journal of Clinical Medicine*.

The authors agreed to this retraction.
